# Ubiquitin-specific Protease 15 Negatively Regulates Virus-induced Type I Interferon Signaling via Catalytically-dependent and -independent Mechanisms

**DOI:** 10.1038/srep11220

**Published:** 2015-06-10

**Authors:** Huan Zhang, Dang Wang, Huijuan Zhong, Rui Luo, Min Shang, Dezhi Liu, Huanchun Chen, Liurong Fang, Shaobo Xiao

**Affiliations:** 1 State Key Laboratory of Agricultural Microbiology, College of Veterinary Medicine, Huazhong Agricultural University, Wuhan 430070, China; 2 The Cooperative Innovation Center for Sustainable Pig Production, Wuhan 430070, China

## Abstract

Viral infection triggers a series of signaling cascades, which converge to activate the transcription factors nuclear factor-κB (NF-κB) and interferon regulatory factor 3 (IRF3), thereby inducing the transcription of type I interferons (IFNs). Although not fully characterized, these innate antiviral responses are fine-tuned by dynamic ubiquitination and deubiquitination processes. In this study, we report ubiquitin-specific protease (USP) 15 is involved in regulation of the retinoic acid-inducible gene I (RIG-I)-dependent type I IFN induction pathway. Knockdown of endogenous USP15 augmented cellular antiviral responses. Overexpression of USP15 inhibited the transcription of IFN-β. Further analyses identified histidine 862 as a critical residue for USP15’s catalytic activity. Interestingly, USP15 specifically removed lysine 63-linked polyubiquitin chains from RIG-I among the essential components in RIG-I-like receptor-dependent pathway. In addition, we demonstrated that in contrast to USP15 de-ubiquitinating (DUB) activity, USP15-mediated inhibition of IFN signaling was not abolished by mutations eliminating the catalytic activity, indicating that a fraction of USP15-mediated IFN antagonism was independent of the DUB activity. Catalytically inactive USP15 mutants, as did the wild-type protein, disrupted virus-induced interaction of RIG-I and IFN-β promoter stimulator 1. Taken together, our data demonstrate that USP15 acts as a negative regulator of RIG-I signaling via DUB-dependent and independent mechanisms.

The infection of viral induces a strong antiviral immune response characterized by robust production of type I interferons (IFNs) and proinflammatory cytokines. Type I IFNs mainly consist of the IFN-β and IFN-α cytokines families, which are the key factors mediating not only the innate immune response but also the subsequent virus-induced development of adaptive immunity^1^. During viral infection, the innate immune defenses are triggered through pathogen-associated molecular patterns (PAMPs)^2^. The sensors of PAMPs, known as pattern-recognition receptors (PRRs), include toll-like receptors (TLRs), the retinoic acid-inducible gene I (RIG-I)-like receptors (RLRs) and nucleotide-oligomerization domain (NOD)-like receptors^3^. The RLRs consist of RIG-I, melanoma differentiation-associated gene 5 (MDA5), and laboratory of genetics and physiology 2 (LGP2), all of which are structurally similar in having the DExD-box RNA helicase domain, and recognize viral RNA^4^. Being the founding PRR member, RIG-I has two N-terminal caspase recruitment domains (CARDs), a DExD/H box helicase/ATPase domain, and a C-terminal repressor domain (CTD)^5^. Upon stimulation, the N-terminal CARDs of RIG-I are responsible for the recruitment and activation of IFN-β promoter stimulator 1 (IPS-1, also known as MAVS/VISA/Cardif) through the CARDs^6^,^7^,^8^,^9^. In turn, IPS-1 activates the downstream adaptor cytosolic protein kinases, including I-κB kinase (IKK) and TANK-binding kinase 1 (TBK1), which respectively activate the transcription factors nuclear factor-κB (NF-κB) and interferon regulatory factor 3 (IRF3), ultimately leading to the production of antiviral mediators such as the type I IFNs and inflammatory cytokines^5^,^10^. IFNs initiate a series of signaling cascades in the Janus kinase/signal transducer and activator of transcription (JAK/STAT) pathway, triggering the expression of a set of IFN-stimulated genes (ISGs), then these genes collaborate to suppress the replication of the virus and contribute to the development of the adaptive immune response 11.

Ubiquitin (Ub) is a 76-residue polypeptide that is highly conserved among eukaryotes. Ubiquitination is a reversible posttranslational modification that involves the covalent attachment of one or more ubiquitin monomers to lysine residues of a target protein, in a process called “monoubiquitylation” or “polyubiquitylation”, respectively^12^,^13^. Emerging evidence indicates that ubiquitin plays a pivotal role in a myriad of biological processes, including protein degradation, cell-cycle regulation, kinase activation, and cell signal transduction^14^. The ubiquitin chains linked *via* lysine (Lys)48 or Lys63 are best uncovered as yet^15^. Indeed, polyubiquitin chains linked through Lys48 tag substrates for degradation by the 26s proteasome. In contrast, those linked through Lys63 are associated with several nondegradative processes, such as endocytosis, DNA repair, protein–protein interactions, and other activities^14^,^16^. Ubiquitination is a reversible process that can be counter-regulated by deubiquitinating enzymes (DUBs), making it ideally suited for controlling the regulation of cellular functions. DUBs can be characterized into five families based on their structural domains, and USPs have been deemed to represent the bulk of DUBs^17^.

Ubiquitination and deubiquitination are critically involved in regulating the virus-induced type I IFN pathway. A vast array of proteins in the signaling cascade are activated by ubiquitination and some ubiquitin ligase enzymes have been reported to play crucial roles, such as RIG-I, TRAF3, TRAF6, and TBK1^18^,^19^. Although the specificities and functions of various deubiquitinases have not been fully characterized, some DUBs that modulate the immune response have been identified. A20 negatively regulates NF-κB activation through the deubiquitination of TRAF2, TRAF6 and RIP1^20^,^21^,^22^. Deubiquitinating enzyme A (DUBA) deconjugates the polyubiquitin chains from TRAF3, leading to its dissociation from TANK-binding kinase 1 (TAK1)^23^. However, the two important regulatory mechanisms in biology, ubiquitination and deubiquitination, need further characterized.

In the present study, we identified USP15 was involved in the downregulation of the production of IFN-β by regulating various signals. To verify the specific mechanism of USP15-mediated IFN inhibition, we identified the DUB activity site His862 of USP15 *in vivo* and *in vitro*, and we presented evidence that USP15 functions as a RIG-I deubiquitinase and specifically removes Lys63-linked polyubiquitin chains. We evaluated the role of USP15 DUB activity in interferon antagonism. By generating dose-response profiles of IFN antagonism, we found USP15 does not strictly require enzymatic activity to inhibit type I IFN expression. Further results proved that USP15 impairs the RIG-I-IPS-1 interaction in a DUB activity-independent manner, supporting the notion of a catalysis-independent mechanism for IFN inhibition. Overall, we identified USP15 as a multifunctional regulator of the virus-induced type I IFN signaling pathway *via* DUB-dependent and independent mechanisms.

## Results

### Knockdown of USP15 results in upregulation of type I IFN

**Figure 1:**
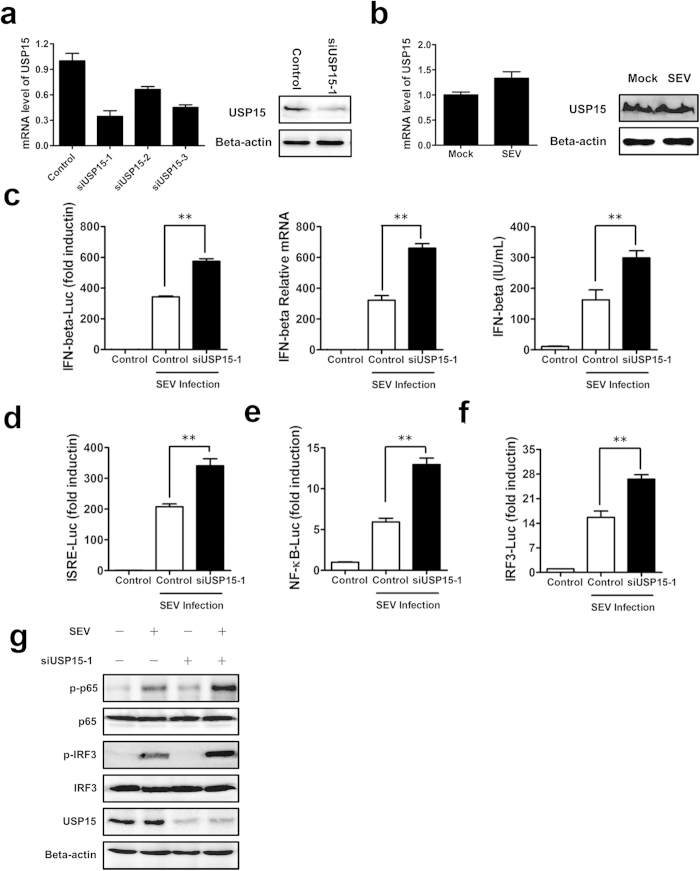
Knockdown of USP15 results in upregulation of type I IFN. (**a**) Knockdown effect of the siRNAs at the mRNA and protein level. HEK293T cells were transfected with USP15-specific siRNAs and the endogenous USP15 mRNA and protein were assayed by real-time RT–PCR and immunoblotting. (**b**) The mRNA and protein level of USP15 after viral infection. HEK293T cells were infected with SEV or mock infected for 16 h. And the endogenous USP15 mRNA and protein were assayed by real-time RT–PCR and immunoblotting. (**c**) Effects of USP15 knockdown on SEV-induced activation of the IFN-β promoter, IFN-β mRNA and protein levels. HEK293T cells were transfected with the IFN-β–Luc reporter plasmid (0.1 μg) and pRL-TK plasmid (0.02 μg), together with plasmid encoding the USP15-specific siRNAs. At 24 h after transfection, the cells were further infected with SEV or mock infected for 16 h before luciferase assays were performed. Meanwhile, the supernatants were collected for ELISA, and the total RNA was then extracted and the expression of *IFNB1* was evaluated with SYBR Green real-time RT–PCR. Data are means ± SD from three independent experiments. ** indicates p ≤ 0.01. (d–f) Effects of USP15 knockdown on the activation of ISRE and the NF-κB and IRF3 promoters. The experiments were performed as in (**c**), except that the ISRE–Luc, NF-κB–Luc, and IRF3–Luc reporter plasmids were used. Data are means ± SD from three independent experiments. (**g**) Effects of USP15 knockdown on SEV-induced phosphorylation of NF-κB subunit p65 and IRF3. HEK293T cells were transfected with USP15-specific siRNAs or control siRNAs for 24 h. Cells were further infected with SEV or mock infected for 16 h before immunoblots with the indicated antibodies were performed.

By using reporter assays in HEK293T cells to screen a pool of 162 siRNAs targeting 54 human USPs genes for the capabilities to modulate SEV-induced activation of the ISRE promoter, we found USP15 is a potential candidate in previous study^24^. To dissect the specific mechanism by which USP15 plays a role in regulating the antiviral innate immune response, we continued to knockdown cell-endogenous USP15 in HEK293T cells to explore the physiological relevance. Since siRNA pools (three siRNAs per gene) were used to conduct the selection in previous study, we firstly evaluated the knockdown effect of each siRNA. As shown in Fig. 1a, siUSP15-1 was found to have a higher efficiency to block USP15 transcription, and the protein level of USP15 was inhibited by siUSP15-1 confirmed through immunoblotting. Thus, siUSP15-1 was used in the following experiments. As displayed in Fig. 1c, the activity of IFN-β report plasmid (342.5 vs 574.5, p = 2.28E-05), transcription of endogenous *IFNB1* gene (321.5 vs 660.0, p = 1.22E-05) and the production of IFN-β proteins (162.4 vs 298.9, p = 2.95 E-05) were higher in the USP15-knocked-down cells than in the control-siRNA-treated cells with Sendai virus (SEV) being the stimulus, under the condition that SEV infection had no effect on USP15 expression (Fig. 1b). Next, we measured the effect of USP15 knockdown on ISRE, an important response element in triggering the expression of ISGs after the activation of IFN^25^. Note that knockdown of USP15 resulted in upregulation of SEV-induced activation of ISRE (207.4 vs 340.9, p = 6.78E-04) (Fig. 1d). Further supporting the notion that USP15 impacts the downstream cascade that amplifies type I IFN expression are the data shown in Supplementary Fig. S1, which suggest that knockdown of USP15 potentiated the SEV-induced expression of ISGs (*ISG15*, *ISG20*, *ISG54*, *ISG56*, *IP-10*).

It is well known that the induction of type I IFN requires the coordinated and cooperative actions of NF-κB and IRF3^10^. Therefore, we investigated whether USP15 is involved in the SEV-induced activation of NF-κB and IRF3. As shown in Fig. 1e and 1f, knockdown of USP15 increased the activation of NF-κB (5.9 vs 12.9, p = 1.92E-04) and IRF3 (15.6 vs 26.5, p = 1.04E-03) through reporter assays. To further confirm the results, SEV-induced phosphorylation of NF-κB subunit p65 and IRF3 were enhanced by the knockdown of USP15 (Fig. 1g). Overall, these data showed that knockdown of cellular USP15 has an enhanced effect on the type I IFN signaling pathway.

### Overexpression of USP15 suppresses the virus-induced type I IFN signaling pathway

**Figure 2:**
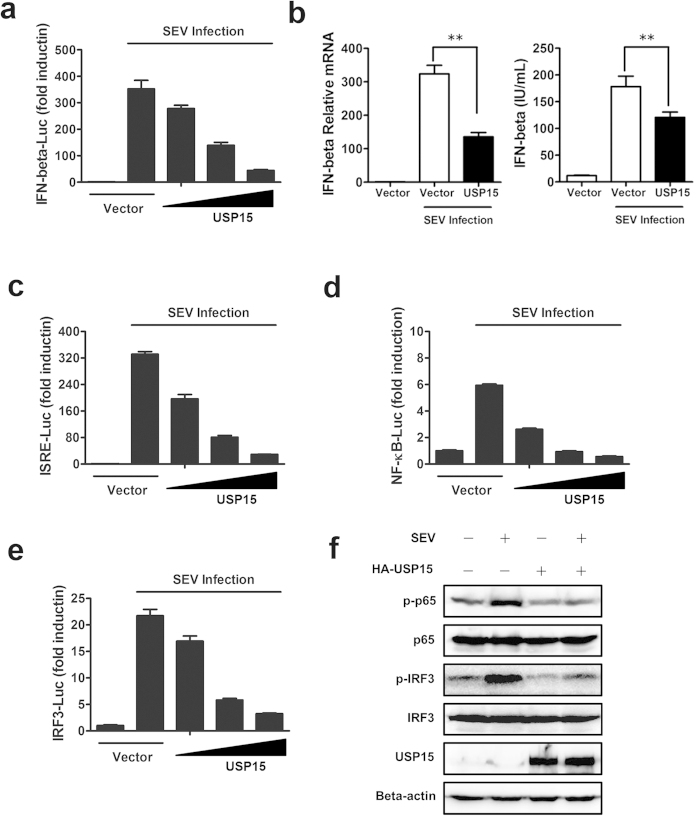
USP15 suppresses the virus-induced type I IFN signaling pathway. (**a**) USP15 inhibited the SEV-induced activation of the IFN-β promoter. HEK293T cells grown in 24-well plates were transfected with the IFN-β–Luc reporter plasmid (0.1 μg) and pRL-TK plasmid (0.02 μg) together with increasing quantities (0, 0.1, 0.3, or 0.9 μg) of plasmid encoding USP15. At 24 h after transfection, the cells were further infected with SEV or mock infected for 16 h before luciferase assays were performed. Data are means ± SD from three independent experiments. (**b**) USP15 inhibited SEV-induced IFN-β mRNA and protein levels. HEK293T cells were transfected with plasmid encoding USP15 (1 μg) or an equivalent amount of empty vector. Then the experiments were performed as in (Fig. 1c). Data are means ± SD from three independent experiments. ** indicates p ≤ 0.01. (c–e) USP15 inhibited SEV-induced activation of ISRE and the NF-κB and IRF3 promoters. The experiments were performed as in (**a**), except that the ISRE–Luc, NF-κB–Luc, and IRF3–Luc reporter plasmids were used. Data are means ± SD from three independent experiments. (f) USP15 inhibited SEV-induced phosphorylation of NF-κB subunit p65 and IRF3. HEK293T cells were transfected with plasmid encoding USP15 (3 μg) or an equivalent amount of empty vector for 24 h, and then the cells were infected with SEV or mock infected for 16 h. An immunoblotting analysis was performed with the indicated antibodies.

The observation that depletion of USP15 resulted in upregulated production of IFN prompted us to further examine the effects of USP15 overexpression on type I IFN signaling pathway. To this end, HEK293T cells were transfected with an expression construct encoding USP15, together with a luciferase reporter plasmid containing the IFN-β promoter and pRL-TK and cells were further stimulated with SEV. The result showed that USP15 caused the dose-dependent inhibition of IFN-β promoter activity (Fig. 2a). Coincident with knockdown experiments, overexpression of USP15 blocked SEV-induced transcription of endogenous *IFNB1* gene (323.8 vs 135.4, p = 1.0E-04) and the production of IFN-β proteins (178.2 vs 120.6, p = 3.52E-04) (Fig. 2b). Similarly, we observed that increasing quantities of USP15 caused the inhibition of SEV-induced activation of ISRE (Fig. 2c), NF-κB (Fig. 2d), and IRF3 (Fig. 2e) promoters, additionally, the expression of ISGs (Supplementary Fig. S1). We also determined whether USP15 is involved in regulation of virus-induced IFN signaling in other cell type. As observed in HEK293T cells, ectopic expression of USP15 inhibits SEV-induced IFN-β promoter activation, while knockdown of USP15 by siRNA results in upregulation of the activation of IFN-β, NF-κB and IRF3 promoters in A549 cells, as well as the production of *IFNB1* and ISGs (Supplementary Fig. S2). Decrease of SEV-induced phosphorylation of NF-κB subunit p65 and IRF3 (Fig. 2f) were also observed in USP15 overexpression cells. These overexpression experiments results together with those of knockdown results highlight that USP15 is involved in virus-induced type I IFN signaling by serving as a potent inhibitor.

### Identification of USP15 DUB activity site His286

**Figure 3:**
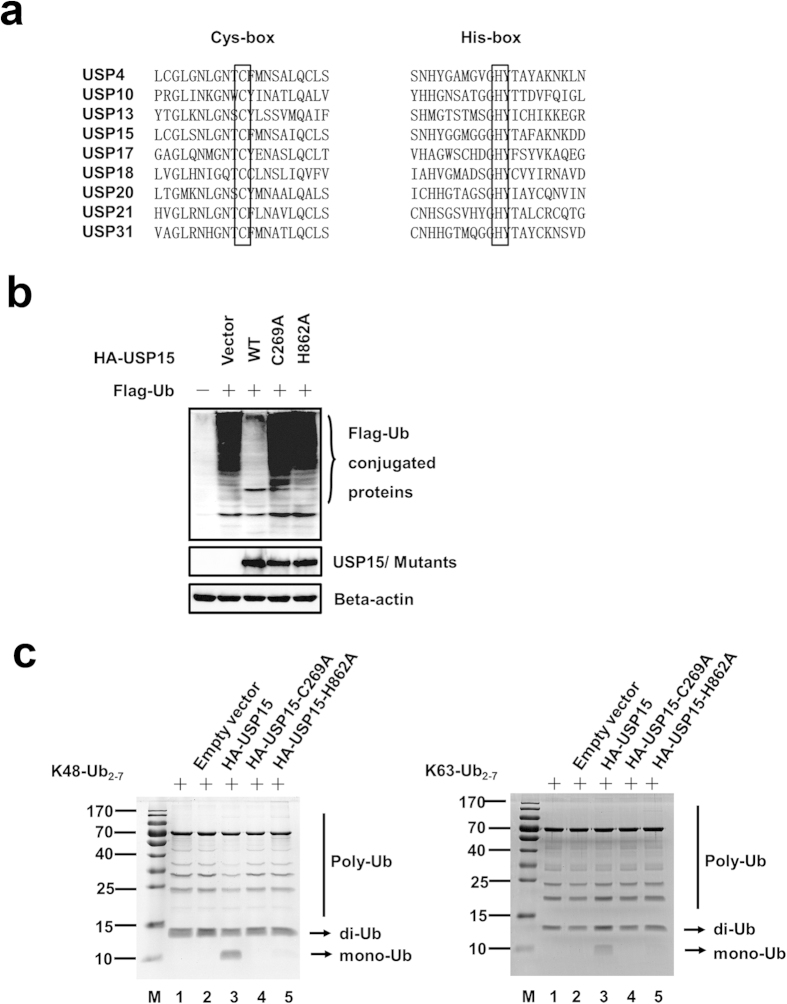
Identification of the DUB activity site His862 *in vivo and in vitro*. (**a)** Putative sites responsible for the DUB activity of USP15. Black boxes indicate the conserved residues tested in this experiment. The sequences were taken from GenBank entries with the following accession numbers: USP4, NM_003363; USP10, NM_001272075; USP13, NM_003940; USP15, NM_006313; USP17, NM_201402 and XM_352721; USP18, NM_017414; USP20, NM_001110303; USP21, NM_001014443; and USP31, NM_020718. (**b**) H862 is the catalytic site. HEK293T cells were co-transfected with a Flag-tagged Ub expression plasmid (1.0 μg) and expression vectors encoding HA–USP15-WT, HA–USP15-C269A, or HA–USP15-H862A. The immunoblotting analysis was performed with anti-HA and anti-Flag antibodies. (**c**) Identification of the DUB activity *in vitro*. The proteins were obtained from USP15/USP15-C269A/USP15-H862A-transfected or mock-transfected HEK293T cells using the HA tagged Protein PURIFICATION KIT. Lys48-/Lys63-linked polyubiquitin was incubated with the protein obtained from mock-transfected (lane 2), USP15-transfected (lane 3), USP15-C269A-transfected (lane 4) or USP15-H862A-transfected (lane 5) HEK293T cells at 37 °C for 30 min, then analyzed by SDS–PAGE. Lane 1, uncleaved Lys48-linked polyubiquitin chain (K48-Ub_2–7_).

Most DUBs exhibit a high degree of amino acid homology, predominantly in the two regions known as the cysteine (Cys) box and the histidine (His) box, which surround the catalytic Cys and His residues, respectively, and have been reported to be essential for the catalytic properties of the USPs^26^. We chose eight USPs with previously documented DUB activity^27^,^28^,^29^,^30^,^31^,^32^,^33^, and a sequence alignment showed that Cys269 and His862 of USP15 are highly conserved among all nine USPs (Fig. 3a). Since the DUB activity of C269 has been identified^34^,^35^, we tried to test whether the deubiquitinase activity of USP15 is dependent on intact catalytic residues H862, with C269 being the positive control. As shown in Fig. 3b, mutations at both sites abrogated the deubiquitinase activity of the mutant proteins. Fig. 3c presents data that further support the notion that the USP15 mutants had no deubiquitinase activity for Lys48- or Lys63-linked ubiquitin *in vitro*. Therefore, the catalytic activity of USP15 is dependent on His862.

### USP15 specifically deconjugates the ubiquitination of RIG-I

**Figure 4:**
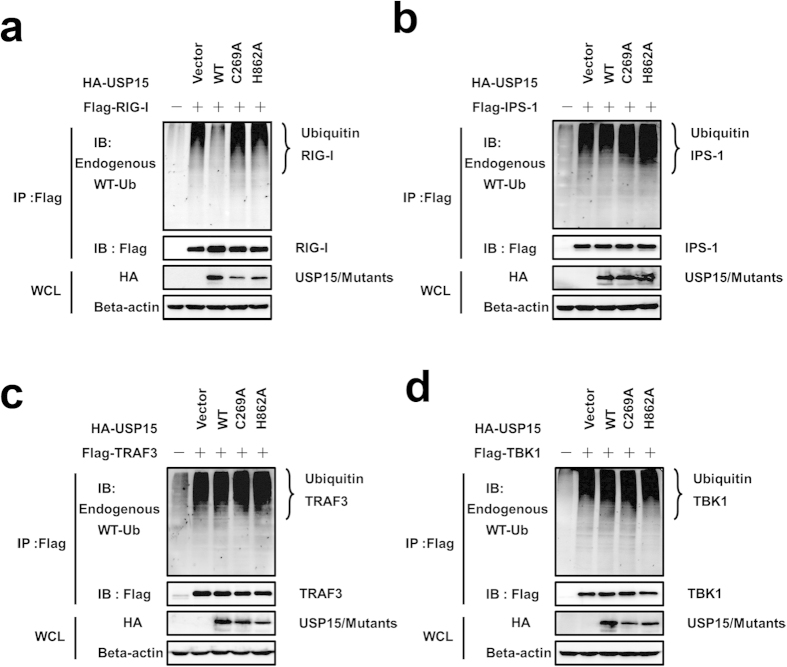
USP15 specifically deconjugates the ubiquitination of RIG-I. (**a–d**) HEK293T cells were co-transfected with Flag-tagged RIG-I, IPS-1, TRAF3, TBK1 and HA–USP15-WT or a catalytic mutant (USP15-C269A or USP15-H862A). The cell lysates were immunoprecipitated with an anti-Flag antibody and immunoblotted with an anti-Ub antibody.

RIG-I-dependent signaling pathway plays a vital role in the recognition of virus-derived double-stranded RNAs and the induction of type I IFN^36^. During the innate antiviral immune response, ubiquitination of essential signaling components is pivotal in signal transduction^37^. Therefore, we determined whether USP15 acts as a DUB targeting RIG-I-like receptor-mediated IFN signaling. Toward this objective, HEK293T cells were co-transfected with Flag-tagged RIG-I, IPS-1, TRAF3, TBK1 and hemagglutinin (HA)–USP15-WT or a catalytic mutant (USP15-C269A or USP15-H862A), and the endogenous ubiquitination of proteins were assessed with an anti-Ub antibody. The overexpression of USP15-WT, but not of the deubiquitinase-deficient mutants (USP15-C269A and USP15-H862A), significantly decreased the ubiquitination of RIG-I (Fig. 4a) and did not block ubiquitination of IPS-1 (Fig. 4b), TRAF3 (Fig. 4c) and TBK1 (Fig. 4d), illustrating that USP15 has deubiquitination activity for RIG-I.

### USP15 removes Lys-63 linked polyubiquitination conjugates from RIG-I

**Figure 5:**
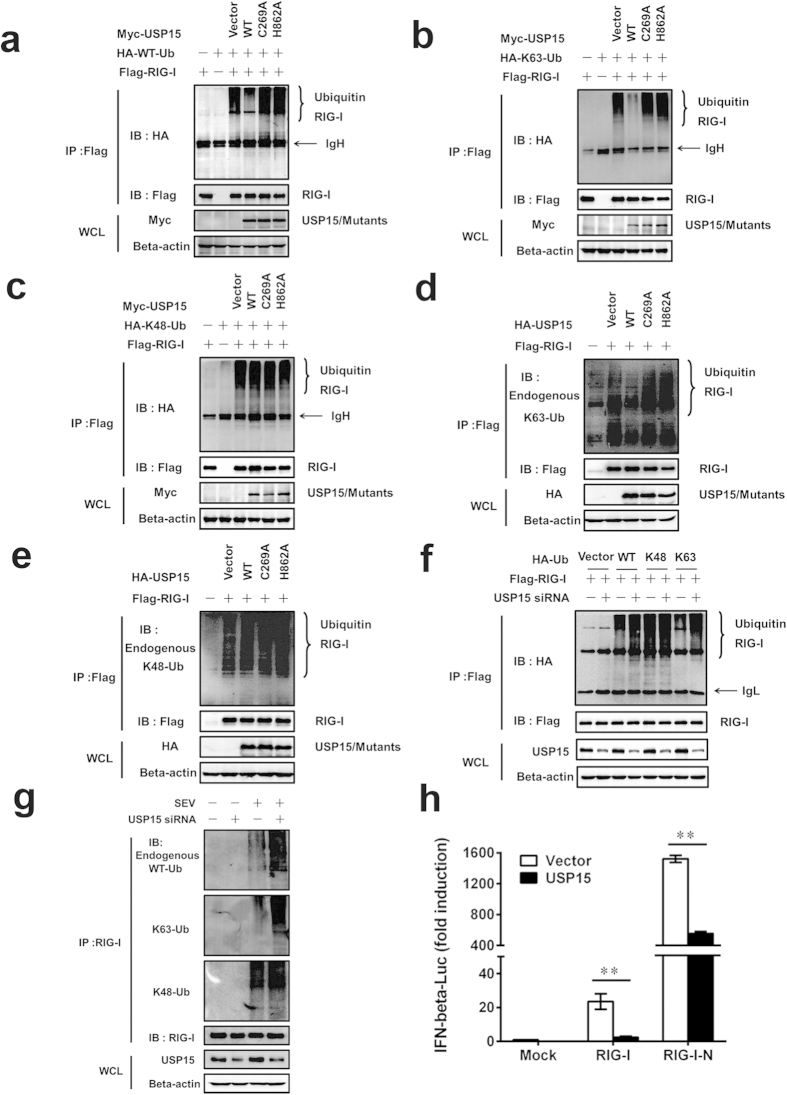
USP15 deconjugates Lys63-linked polyubiquitin chains from RIG-I. (**a–c**) HEK293T cells were cotransfected with expression vectors encoding Flag–RIG-I and HA–WT-Ub (**a**), Flag–RIG-I and HA–K63-Ub (**b**), or Flag–RIG-I and HA–K48-Ub (**c**), together with control vectors or expression vectors encoding Myc–USP15-WT, Myc–USP15-C269A, or Myc–USP15-H862A. The cell lysates were immunoprecipitated with an anti-Flag antibody and immunoblotted with an anti-HA antibody. (**d–e**) HEK293T cells were co-transfected with an expression vector encoding Flag–RIG-I and control vectors or expression vectors encoding HA–USP15-WT or the mutant HA–USP15-C269A or HA–USP15-H862A. The cell lysates were immunoprecipitated with an anti-Flag antibody and immunoblotted with an anti-Ub (K63) antibody (**d**), or anti-Ub (K48) antibody (**e**). (**f**) Knockdown of USP15 potentiated the ubiquitination of RIG-I. HEK293T cells were transfected with USP15-specific siRNAs or control siRNAs and indicated expression plasmids. The cell lysates were immunoprecipitated with an anti-Flag antibody and immunoblotted with an anti-HA antibody. (**g**) Knockdown of USP15 potentiated SEV-induced ubiquitination of RIG-I. HEK293T cells were transfected with USP15-specific siRNAs or control siRNAs, the cells were then further infected with SEV or mock infected for 16 h. The cell lysates were immunoprecipitated with an anti-RIG-I antibody and immunoblotted with indicated antibodies. (**h**) USP15 inhibited RIG-I- and RIG-I-N-mediated activation of the IFN-β promoter. HEK293T cells were cotransfected with the IFN-β–Luc reporter plasmid (0.1 μg), pRL-TK plasmid (0.02 μg), and 0.5 μg of plasmid encoding USP15, together with the RIG-I or RIG-I-N expression vector (0.5 μg). Luciferase assays were performed 30 h after transfection. Data are means ± SD from three independent experiments. ** indicates p ≤ 0.01.

To identify the forms of USP15-mediated deubiquitination of RIG-I, HEK293T cells were transfected with HA–WT-Ub, HA–Lys (K)48-Ub or HA–K63-Ub together with Flag-RIG-I and Myc–USP15-WT or a catalytic mutant (USP15-C269A or USP15-H862A). Consistent with the negative regulatory role of USP15 in RIG-I-mediated signaling, USP15 substantially reduced RIG-I polyubiquitination in cells transfected with plasmid encoding the WT ubiquitin and Lys63 mutant (Fig. 5a,b), but not in the mutant-Lys48-transfected cells (Fig. 5c), indicating that USP15 has DUB activity directed specifically towards the Lys63-linked polyubiquitin chains of RIG-I. To confirm these results, HEK293T cells were transfected with Flag-tagged RIG-I in the presence of USP15-WT or the mutants, and the proteins were detected with indicated antibodies. The data showed that USP15 markedly deubiquitinated the Lys63-linked endogenous ubiquitin chains from RIG-I (Fig. 5d–e). To further confirm USP15 deubiquitinates polyubiquitination of RIG-I, USP15 siRNAs were transfected into HEK293T cells to knockdown endogenous USP15 expression. Coimmunoprecipitation analysis showed that compared with control siRNA transfected cells, knockdown of USP15 substantially increased RIG-I polyubiquitination in WT ubiquitin and mutant Lys63 transfected cells (Fig. 5f). As shown in Fig. 5g, USP15 knockdown greatly enhanced SEV-induced WT and K63-linked polyubiquitination of RIG-I, further confirming USP15-mediated deubiquitination of RIG-I under physiological conditions. In conclusion, our results provide clear evidence that USP15 deconjugates the Lys63-linked polyubiquitin chains from RIG-I.

Since the activation of RIG-I signaling requires Lys63-linked ubiquitination, then we investigated whether the overexpression of USP15, which removes the Lys63-linked polyubiquitin from RIG-I, will decreases the RIG-I-mediated activation of IFN-β promoter activity and the activation mediated by its constitutively active mutant, RIG-I-N. As shown in Fig. 5h, the overexpression of RIG-I or RIG-I-N significantly activated the IFN-β promoter compared with that in cells transfected with the empty vector. However, these effects were all markedly reduced in the presence of USP15 (23.5 vs 2.5, p = 1.39E-04; 1522.8 vs 554.6, p = 5.74E–04). In addition, USP15 dose-dependently inhibited RIG-I-N-triggered IFN-β–Luc, ISRE–Luc, NF-κB–Luc, and IRF3–Luc reporter activities (Supplementary Fig. S3). These findings taken together support the hypothesis that USP15 function as a negative regulator of IFN signaling.

### Mutation of the catalytic residues does not completely abolish USP15 IFN antagonism

**Figure 6:**
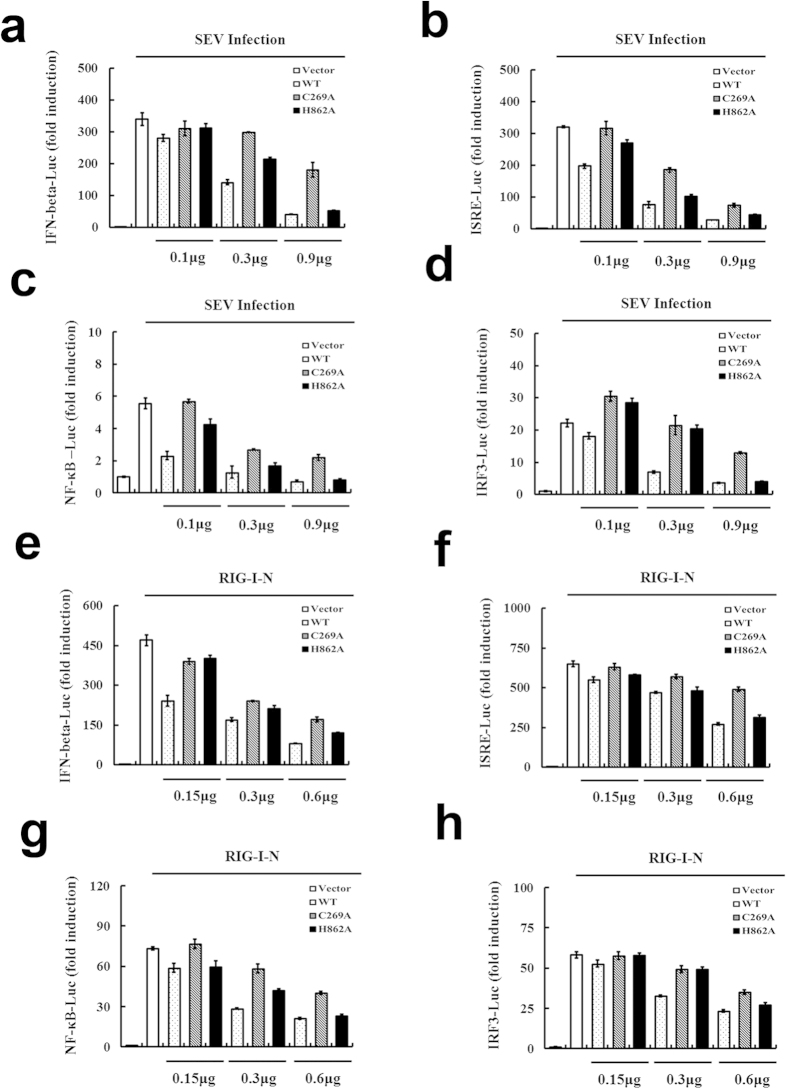
Mutation of the catalytic residues does not abolish USP15 IFN antagonism. (**a-d**) HEK293T cells were co-transfected with increasing amounts of plasmid encoding USP15 or the catalytic mutant USP15–C269A or USP15–H862A, together with different reporter plasmids (0.1 μg) and the pRL-TK plasmid (0.02 μg) for 24 h. The cells were then infected with SEV or mock infected for 16 h before the luciferase assays were performed. Data are means ± SD from three independent experiments. (**e–h**) HEK293T cells were co-transfected with increasing amounts of plasmid encoding USP15 or the USP15-C269A or USP15-H862A catalytic mutant, together with different reporter plasmids (0.1  μg), pRL-TK plasmid (0.02  μg), and plasmid encoding RIG-I-N. Luciferase assays were performed 30  h after transfection. Data are means ± SD from three independent experiments.

We also assessed whether USP15 deubiquitinase activity is required for its inhibitory role in SEV-induced IFN induction. To this end, HEK293T cells were transfected with expression vectors encoding the catalytic mutants of USP15 (USP15-C269A or USP15-H862A) or USP15-WT, together with IFN-β, ISRE, NF-κB or IRF3 reporter plasmids. We found that, like the USP wild type, both USP15-C269A and USP15-H862A exhibit dose-dependent inhibition of activities of the four reporter plasmids, not completely abrogated the IFN antagonism capability (Fig. 6a–d). Remarkably, the antagonism profile of USP15-H862A is similar to that of USP15-WT. Similar results were also obtained when RIG-I-N was used in lieu of SEV to compare the inhibitory effects of the mutants and wild-type USP15 (Fig. 6e–h). Through real-time RT-PCR, we also found that USP15-WT, USP15-C269A and USP15-H862A inhibit the transcription of endogenous *IFNB1* gene and the SEV-induced expression of ISGs (Supplementary Fig. S4). These results indicate that catalytic activity of USP15 is not strictly required for inhibition of antiviral IFN expression.

### USP15 sequesters the interaction between RIG-I and IPS-1 in a DUB activity-independent manner

**Figure 7:**
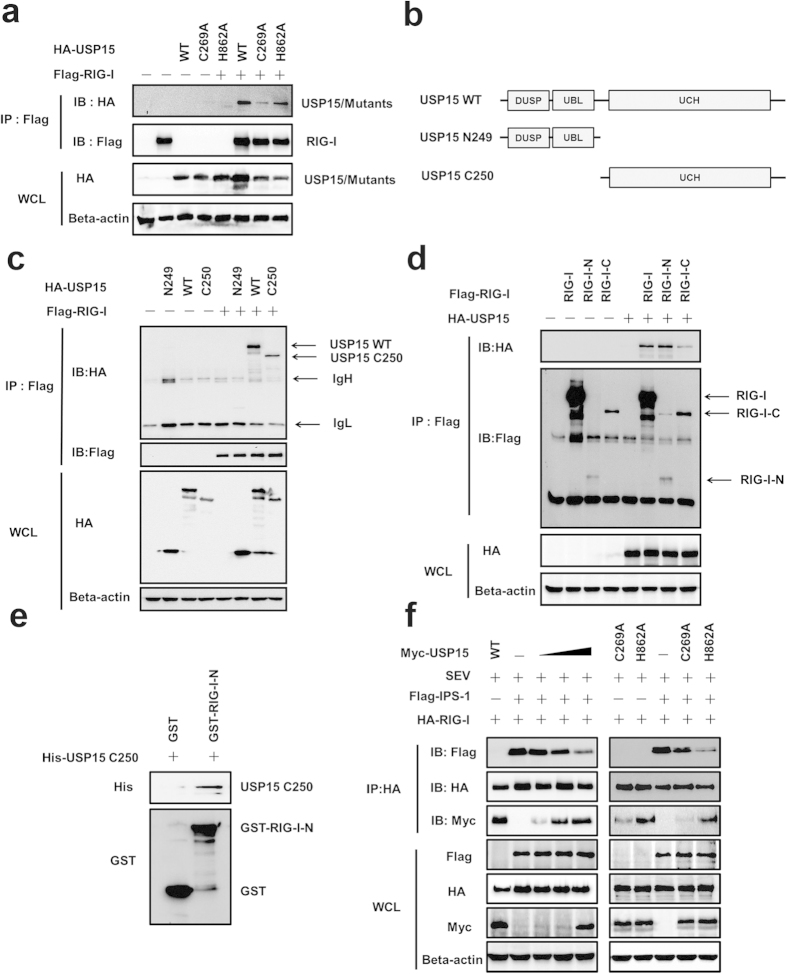
USP15 sequesters the interaction between RIG-I and IPS-1 in a DUB activity-independent manner. (**a**) The interaction between USP15 and RIG-I was independent of the DUB activity of USP15. HEK293T cells were co-transfected with expression vectors encoding HA–USP15-WT or the mutant HA–USP15-C269A or HA–USP15-H862A, together with control vectors, or expression vectors encoding Flag–RIG-I. The cell lysates were immunoprecipitated with an anti-Flag antibody and immunoblotted with an anti-HA antibody. (**b**) Schematic diagram of the deletion mutants of USP15 used in this study. (**c**) The UCH domain of USP15 was responsible for its interaction with RIG-I. HEK293T cells were co-transfected with plasmid encoding wild-type HA–USP15 or one of its deletion mutants, together with control vectors or expression vectors encoding Flag–RIG-I. The cell lysates were immunoprecipitated with an anti-Flag antibody and immunoblotted with an anti-HA antibody. (**d**) The N-terminal CARD domain and the C-terminal domain of RIG-I both bind to USP15. HEK293T cells were co-transfected with plasmid encoding wild-type Flag–RIG-I or one of its deletion mutants, together with control vectors or expression vectors encoding HA–USP15. The cell lysates were immunoprecipitated with an anti-Flag antibody and immunoblotted with an anti-HA antibody. (**e**) GST pulldown assay to confirm the interaction between RIG-I and USP15. Glutathione beads conjugated to GST or GST-RIG-I-N (from recombinant E. coli) were incubated with purified USP15 C250. After washing five times, the beads were boiled and subjected to immunoblotting. (**f**) USP15 sequestered the interaction between RIG-I and IPS-1. HEK293T cells were co-transfected with plasmid encoding HA–RIG-I, Flag-IPS-1, increasing amounts of wild-type Myc–USP15 or its mutants. 24 h later, cells were further infected with SEV. The cell lysates were immunoprecipitated with an anti-HA antibody and immunoblotted with anti-Flag and anti-Myc antibodies.

Because the catalytic mutants of USP15 did not completely lose the ability to block type I IFN signaling, especially USP15-H862A still exhibited great potential to act as a potent inhibitor. We wondered whether these mutants, lacking DUB activity, were still capable of interacting with RIG-I. Therefore, HA-tagged USP15-WT or the mutants (USP15-C269A or USP15-H862A) together with Flag-tagged RIG-I were transfected into HEK293T cells. The cell lysates were immunoprecipitated with an anti-Flag antibody, and the presence of HA-tagged proteins was assessed by immunoblotting. In this assay, we found that Flag–RIG-I coimmunoprecipitated with HA–USP15-WT, as well as the USP15-C269A and USP15-H862A mutants (Fig. 7a). In addition, compared with USP15-C269A, USP15-H862A showed stronger interaction with RIG-I, suggesting that substituting the cysteine residue with an alanine may impair the combination with RIG-I.

We also examined the specificity of the association between USP15 and RIG-I. USP15 is composed of an N-terminal DUSP domain and a C-terminal UCH domain. We used a coimmunoprecipitation experiment to map the domain of USP15 that facilitates its interaction with RIG-I. As demonstrated in Fig. 7c, RIG-I interacted with USP15-WT and the C-terminal UCH domain (USP15-C250), but not with the N-terminal DUSP domain (USP15-N249). We also identified the domains of RIG-I responsible for the RIG-I–USP15 interaction. RIG-I has an N-terminal domain consisting of two CARD motifs and a C-terminal domain. A coimmunoprecipitation assay suggested that mutants carrying the N-terminal CARD domain or the C-terminal domain both interacted with USP15 (Fig. 7d). Together, these results demonstrate that USP15 and RIG-I form a complex through the UCH domain of USP15, and that the N-terminal CARD domain and the C-terminal domain of RIG-I both bind to USP15. To further confirm the interaction *in vitro*, we employed glutathione S-transferase (GST) pull down assays by using the purified USP15 C250 (from recombinant *E. coli*) and glutathione beads conjugated with GST-RIG-I-N or GST protein. Our result showed that the presence of GST-RIG-I-N retained USP15 C250 (Fig. 7e), indicating that the interaction of RIG-I with USP15 is due to a direct physical association.

The production of type I IFN requires the recruitment of IPS-1 to the N-terminal CARDs of RIG-I^6^. Because our results demonstrated that USP15 interacts with RIG-I-N, it is reasonable to infer that USP15 is an alternative substrate of RIG-I, which impairs the recruitment of IPS-1 by RIG-I. To test this hypothesis, HEK293T cells were transfected with increasing amounts of HA-tagged USP15-WT or the mutants (USP15-C269A or USP15-H862A), together with indicated plasmids. As shown in Fig. 7f, RIG-I efficiently pulled down IPS-1. However, the amount of IPS-1 that bound to RIG-I gradually decreased as the amount of USP15-WT increased, suggesting that USP15 physically sequester the RIG-I-IPS-1interaction in a concentration-dependent manner. Of note, USP15-H862A significantly impaired the interaction between RIG-I and IPS-1. In stark contrast, USP15-C269A exhibited weaker interaction with RIG-I, resulting in a weak reduction of the RIG-I-IPS-1combination. These results together with those of Fig. 6 indicated that suppression of IFN expression was positively correlated with the disruption of RIG-I-IPS-1interaction, highlighting the notion of catalytic-activity-independent mechanism of IFN antagonism.

## Discussion

For serving as an important part of the host defense system, the type I IFN signaling pathway must be tightly controlled to sustain homeostatic immune responses. Ubiquitination and deubiquitination are associated with the modulating virus-triggered induction of type I IFNs. Since RIG-I is a critical PRR in the virus-triggered type I IFN signaling pathway, the function of RIG-I ubiquitination is well established and some regulators have been identified. The ubiquitin E3 ligases TRIM25^5^,^38^, Riplet^39^,^40^ attach Lys63-linked polyubiquitin chains to RIG-I, which stabilize the interaction between RIG-I and IPS-1, thus promoting the activation of the RIG-I signaling pathway. Another E3 ligase, RNF125, conjugates Lys48-linked ubiquitin to RIG-I to mediate its proteasomal degradation, acting as a negative regulator^41^. Some DUBs that mediate the deubiquitination of RIG-I have also been defined. CYLD interacts with RIG-I and removes Lys63-linked polyubiquitin chains from it, which might be a mechanism involved in the downregulation of the IFN pathway^42^. It is noteworthy that some USPs, which constitute the largest subfamily of DUBs, are critical players in regulating the RIG-I-mediated innate immune response, including USP4^43^ , USP17^29^ and USP21^44^. USP4 positively regulates the RIG-I-mediated antiviral response through the deubiquitination of Lys48-linked polyubiquitin from RIG-I^43^. USP17 negatively modulates virus-induced type I IFN signaling through the deubiquitination of RIG-I and MDA5^29^. USP21 negatively regulates antiviral response by acting as a RIG-I deubiquitinase^44^. In our study, we found that USP15 specially removes Lys63-linked ubiquitin chains from RIG-I. However, we found that USP15 interferon antagonism is enhanced by, but is not strictly dependent on the catalytic activity. Inhibition of DUB activity by mutagenesis did not abrogate interferon antagonism. Additionally, the expression of RNF125^41^ is induced and the expression of USP4^43^ is reduced after viral infection, whereas, USP15 was not affected. Therefore, we guess USP15 functions as a negative feedback molecule to attenuate the establishment of an antiviral state, in a similar fashion with CYLD^42^, not functioning as an element that is removed to allow the enhancement of response. To sum up, USP15 is a novel modulator of RIG-I signaling pathway.

The results of our study suggest that USP15 has deubiquitinase activity *in vitro* and *in vivo*, and acts on both Lys48- and Lys63-linked polyubiquitin chains. However, USP15 specifically deconjugates Lys63-linked polyubiquitin from RIG-I, resulting in the disruption of downstream signaling and the balanced production of IFN. USP25 is a similar deubiquitinating enzyme that can disassemble both Lys48- and Lys63-linked ubiquitin oligomers *in vitro*, but specifically removes Lys48-linked ubiquitin molecules from TRAF3 and Lys63-linked ubiquitin from TRAF6^45^,^46^,^47^,^48^. We speculate that *in vivo*, different signals in the pathway lead to specific substrates of USP15. Therefore, further investigation is required to confirm our hypothesis.

Using reporter assays, we found that blocking the DUB activity by mutagenesis did not completely abrogate the ability of USP15 to inhibit the type I IFN signaling pathway (Fig. 6). The catalytic mutants USP15-C269A and -H862A, especially USP15-H862A, still dose-dependently inhibited the IFN-β–Luc, ISRE–Luc, NF-κB–Luc, and IRF3–Luc reporter activities. Reminding us USP15 may antagonize type I IFN induction independently of catalytic activity. We hypothesized that USP15 interacts with RIG-I and the interaction is independent of the DUB activity, and the coimmunoprecipitation assay proved the credibility. Mechanistically, the interaction between RIG-I and USP15 suggests the model that USP15 prevents the access of a downstream target to the RIG-I complex. In this study, we noted that IPS-1 was dislodged from RIG-I as the level of USP15 increased (Fig. 7f left). Whereas, the weaken interaction effect suggests two possible models. One possibility is that USP15 deubiquitinates the Lys63 linked polyubiquitination from RIG-I, thus disrupting the recruitment of IPS-1. The second possibility is that USP15 interacts with RIG-I to reduce IPS-1-RIG-I binding. In the same experiment (Fig. 7f right), we found catalytically inactive mutants sequester the RIG-I-IPS-1interaction; however, USP15-H862A, which showed stronger interaction with RIG-I, caused a more profound reduction in RIG-I-IPS-1interaction. These results suggest that USP15 sequesters the interaction between RIG-I and IPS-1 in a DUB activity-independent manner by acting as a competitor of IPS-1 for RIG-I binding, resulting in the inhibition of both IPS-1 activation and subsequent type I IFN production. Additionally, due to the strong inhibition of RIG-I-IPS-1interaction, USP15-H862A exhibited more profound inhibition of IFN induction compared with USP15-C269A (Fig. 6), which corroborates the notion of a catalysis-independent mechanism for type I IFN antagonism. Although we cannot rule out the possibility that USP15 exerts its effects in a third uncharacterized manner, the data in this study demonstrate that USP15 sequesters the interaction between RIG-I and IPS-1, shedding light on the mechanisms underlying the catalytic-activity-independent antagonism of IFN by USP15.

USP15 has been studied extensively in recent years and a growing body of data concerning its functions has been reported. Schweitzer *et al.* reported that COP9 signalosome (CSN)-associated USP15 can deubiquitinate IκBα and regulates the activation of NF-κB^49^. However, Sun *et al.* found no interaction between USP15 and IκBα, and proposed that USP11 inhibits the ubiquitination and degradation of IκBα in the early stage, whereas USP15 functions at a later time point in the TNFα-induced NF-κB activation^50^. USP15 also acts as a key component of the transforming growth factor β (TGF-β) signaling pathway^35^, and is a DUB for R-SMADs^34^. The DUB activity of USP15 is reported to be involved in the regulation Tip110 protein degradation^51^, parkin-mediated mitochondrial ubiquitination and mitophagy^52^, and ALK3/BMPR1A in bone morphogenetic protein signaling^53^. Based on the insights into the characteristics of USP15 gained so far, we can see that USP15 is a multifunctional protein by acting as a DUB for many molecules in different signaling pathway. However, in our study, we found the DUB activity of USP15 is not strictly required for inhibition of antiviral IFN expression, and the effect to disrupt the RIG-I-IPS-1interaction plays a central role in the regulation of IFN signaling pathway. Remarkably, we identified another DUB activity site His862. Despite the fact that both catalytically inactive mutants lost the ability to reduce the ubiquitination of RIG-I (Fig. 5), they showed different potential to downregulate the expression of IFN (Fig. 6). We hold the hypothesis that changing the cysteine residue to an alanine may significantly impair the capability to combine with RIG-I, thus causing a weaker influence on the recruitment of IPS-1 triggered by RIG-I. Even though Cys269 and His862 are both indispensable to DUB activity, the precise role of each site in determining the protein structure is unclear, so there is a high possibility that these two sites determine different binding specificity. Further investigation is required to confirm our hypothesis.

When our work is finished, Pauli and coworkers found that USP15 deubiquitinates the Lys48-linked ubiquitylation of TRIM25 to sustain its stabilization, thus leading to an enhanced production of type I IFN^54^. To determine the precise role of USP15 plays in mediating the IFN signaling pathway and figure out the factors that drive the main different results, we asked for the USP15-Myc and TRIM25-V5 plasmids from Dr. Gack and performed related experiments. We found both USP15-HA and USP15-Myc dose-dependently inhibited the SEV- and RIG-I-N-induced activation of IFN-β (Supplementary Fig. S5). We further investigated the effect of both USP15 expression plasmids on antiviral responses by monitoring the VSV-GFP expression under fluorescence microscope. As shown in supplementary Fig. S5, neither USP15-HA nor USP15-Myc suppressed the replication of VSV, not in accordance with the result described in Pauli’s paper that USP15 limited the VSV replication. In contrast, poly(I:C)-induced inhibition of VSV replication was recovered by overexpression of both USP15-HA and USP15-Myc (Supplementary Fig. S5). These results together suggested that both USP15 constructed in two different expression plasmids inhibited the production of IFN. However, we also found that co-expression of increasing amounts of USP15-HA and USP15-Myc further enhanced the IFN-β promoter activation stimulated by RIG-I-N and TRIM25 as a function of the dose, in line with the result described by Pauli (Supplementary Fig. S5). Through the comparison we found under the condition that co-expressed with TRIM25, USP15 enhanced the activation of IFN-β. However, when expressed alone, USP15 inhibited the IFN signaling pathway. Thus, overexpression of TRIM25 may change the real properties and effects of USP15 in regulating the IFN signaling pathway. To exclude the possibility that these results were attributable to the nonphysiological overexpression of USP15 or TRIM25, we knocked down cell-endogenous USP15 in HEK293T cells and to explore the physiological relevance. Pauli found the ubiquitylation of endogenous RIG-I was reduced in cell line in which USP15 was stably knocked down. To eliminate the diversity between different cell lines, we preferentially performed transient transfection with siRNAs to dissect the biological effect of USP15 towards RIG-I ubiquitination, the results (Fig. 5f,g) collaborate to prove the USP15-mediated deubiquitination of RIG-I. Besides the function of DUB activity in regulating IFN production, USP15 is a competitor of IPS-1 for RIG-I binding, leading to the limitation of the IPS-1 activation and subsequent type I IFN production. So disruption of RIG-I-IPS-1interaction also makes USP15 a negative regulator of IFN pathway. To sum up, USP15 is a potential novel factor maintaining the delicate balance of the virus-induced type I IFN signaling pathway. Being a multifunctional protein, the comprehensive and detailed roles require further researches.

## Methods

### Cells and transfection

HEK293T cells were cultured in RPMI-1640 (HyClone) supplemented with 10% heated-inactivated fetal bovine serum at 37 °C in a humidified 5% CO_2_ incubator. The cells were transfected with expression plasmids using Lipofectamine 2000 (Invitrogen) according to the manufacturer’s instructions.

### Plasmids

Full-length HA-tagged Ub mutants, in which all but one Lys residue (HA–K48-Ub or HA–K63-Ub) was replaced with Arginine (Arg), were the gifts of Tomohiko Ohta (St. Marianna University School of Medicine, Japan)^55^. pcDNA3.1-Flag–Ub and the IPS-1 expression vector were previously described^56^,^57^. The expression plasmids for wild-type RIG-I (pEF-Flag–RIG-I), its constitutively active mutant (pEF-Flag–RIG-I-N), and p125-Luc (IFN-β–Luc) were kindly provided by T. Fujita (Tokyo Metropolitan Institute of Medical Science, Tokyo, Japan)^58^. The luciferase report plasmid (PRDIII-I)_4_–Luc for IRF3 was kindly provided by S. Ludwig (Heinrich Heine University, Düsseldorf, Germany)^59^. The pNF-κB–Luc reporter for NF-κB, the pISRE–Luc reporter for ISRE, and the internal control plasmid phRL-TK were purchased from Stratagene.

The HA or Myc epitope tag was amplified by PCR and cloned into the pCAGGS-MCS^60^ vector to generate the pCAGGS–HA or pCAGGS–Myc plasmid, respectively, encoding an N-terminal HA or Myc tag, respectively. To construct pCAGGS-HA/Myc/USP15, the cDNA fragment encoding full-length USP15 was amplified by PCR (GenBank accession no. NM_006313) and inserted into the pCAGGS–HA or pCAGGS–Myc plasmid. The mutagenesis of individual amino acid residues (C269A and H862A) in USP15 was performed with overlap extension PCR. The detailed sequences of the specific primers used are available upon request. All constructs were validated by DNA sequencing.

### Antibodies and viruses

Mouse monoclonal anti-HA, anti His, anti GST and anti-beta-actin antibodies and rabbit polyclonal anti-USP15, anti-ubiquitin, anti-IRF3, and anti-phosphor-IRF3 antibodies were purchased from ABclonal Biotechnology. Other antibodies used in this study included anti-Flag monoclonal antibody (Macgene, China), anti-Myc monoclonal antibody (Beyotime, China), and anti-NF-κB p65 and anti-phosphor-NF-κB p65 polyclonal antibodies (Cell Signaling Technology). Horseradish-peroxidase-conjugated anti-mouse and anti-rabbit I gG antibodies were purchased from the Beyotime Institute of Biotechnology (Jiangsu, China). SEV was obtained from the Centre of Virus Resource and Information, Wuhan Institute of Virology, Chinese Academy of Sciences. The recombinant VSV expressing GFP (VSV-GFP) was generously provided by Prof. Zhigao Bu, Harbin Veterinary Research Institute, P. R. China.

### Luciferase reporter gene assay

HEK293T cells grown in 24-well plates were transiently co-transfected with 0.1 μg of reporter plasmid (p125–Luc for IFN-β, [PRDIII-I]_4_–Luc for IRF3, pNF-κB–Luc for NF-κB, and pISRE–Luc for ISRE) together with 0.02 μg of phRL-TK plasmid to normalize the transfection efficiency, and various other expression plasmids. The empty vector was used both as the negative control and to adjust the total amount of transfected DNA. In some experiments, the cells were infected with SEV (10 hemagglutinating activity units/well) 24 h after the initial co-transfection and incubated for 16 h. Firefly and *Renilla* luciferase activities were measured using the Dual-Luciferase Reporter Assay System (Promega), according to the manufacturer’s protocol. All reporter assays were repeated at least three times. Data are presented as means ± standard deviations (SD).

### Real-time quantitative reverse transcription (RT)–PCR and enzyme-linked immunosorbent assay (ELISA)

Following the indicated treatment, total cellular RNA was extracted from HEK-293T cells using TRIzol reagent (Invitrogen), after reverse transcription with oligo (dT) primer using a Transcriptor First Strand cDNA Synthesis Kit (Roche), the products were subjected to SYBR green PCR assay (Applied Biosystems) using gene-specific primers designed with Primer Express software v.3.0 (Applied Biosystems). The supernatants from infected cells were collected and subjected to ELISA using a commercial human IFN-β kit (TFB).

### RNA interference

USP15 small interfering RNAs (siRNAs) were synthesized by Sigma as follows: #1: 5´-CUCUUGAGAAUGUGCCGAU-3´; #2: 5´-CACAAUAGAUACAAUUGAA-3´; #3: 5´-CACAUUGAUGGAAGGUCAA-3´. Negative control sequence 5´-UUCUCCGAACGUGUCACGUTT-3´.

### Assay of deubiquitination activity *in vitro*

The USP15 wild-type and catalytic mutants (USP15-C269A and USP15-H862A) proteins were purified from cells transfected with indicated plasmids using the HA tagged Protein PURIFICATION KIT (MBL Japan), according to the manufacturer’s protocol. The polyubiquitin chains K48-Ub_2–7_ (catalogue no. UC-230) and K63-Ub_2–7_ (catalogue no. UC-330) were purchased from Boston Biochem. Purified USP15/USP15-C269A/USP15-H862A (2 μL) was incubated with 3.5 μg of K48–Ub_2–7_ chains or K63–Ub_2–7_ chains at 37 °C in a 14.5 μL reaction mixture containing 25 mM NaCl, 100 μg/mL bovine serum albumin (BSA), and 2 mM dithiothreitol (DTT). A control reaction was incubated under identical conditions without enzyme. The reactions were quenched with the addition of 5 × Sodium dodecylsulphate-polyacrylamide gel electrophoresis (SDS-PAGE) sample loading buffer to a 1 × concentration (25 mM Tris-HCL [pH 6.8], 50% glycerol, 10% SDS, 5% β-mercaptoethanol, 0.2% bromophenol blue) and heat treated at 100 °C for 5 min. The samples were analyzed by electrophoresis on a 12% SDS–PAGE gel and stained with Coomassie Blue.

### Assay of deubiquitination activity in cultured cells

HEK293T cells cultured in 60 mm dishes were co-transfected with 1 μg of Flag–Ub, plus the appropriate amount of construct encoding USP15 or the corresponding mutants, using Lipofectamine 2000. After 28 h, the cells were harvested by adding 200 μL of 2 × lysis buffer A (LBA) (65 mM Tris-HCl [pH 6.8], 4% SDS, 3% DTT, and 40% glycerol) containing 20 mM N-ethylmaleimide (NEM) (Sigma-Aldrich) and 20 mM iodoacetamide (Sigma-Aldrich), and then resolved by 10% SDS–PAGE. The separated proteins were electroblotted onto PVDF membranes (Millipore, Billerica, MA) and the membranes were blocked with 10% (w/v) dried skim milk in tris-buffered saline containing Tween 20 (TBST). The ubiquitin-conjugated proteins were analyzed by immunoblotting with anti-Flag antibody (1:2,000). To confirm the expression levels of USP15 and its mutant forms, an anti-HA antibody was used to detect the HA-tagged proteins. Beta-actin was detected with an anti-beta-actin monoclonal antibody to demonstrate that equal protein samples were loaded. The secondary antibodies (horseradish-peroxidase-conjugated anti-mouse and anti-rabbit I gG antibodies) were used at dilutions of 1:3000, and the protein bands were visualized using the SuperSignal West Pico Luminol kit (Pierce).

### Coimmunoprecipitation and immunoblot analyses

To investigate the interactions of the proteins, coimmunoprecipitation and immunoblotting were performed as follows. HEK293T cells were lysed in 1 mL of lysis buffer (50 mM Tris-HCl [pH 7.4], 150 mM NaCl, 10% glycerol, 1% Nonidet P-40, 0.1% SDS, and 2 mM Na_2_EDTA). After the lysate was collected by centrifugation at 12000 × g for 10 min, the lysate proteins were incubated overnight at 4 °C with 0.5 μg of the indicated antibody; 30 μl of Protein A+G agarose beads (Beyotime, China) was then added to each immunoprecipitation reaction for another 6 h. The agarose beads were then washed three times with 1 mL of lysis buffer. The precipitates were subjected to immunoprecipitation with an anti-Flag antibody, and the captured proteins were detected by immunoblotting with the indicated antibodies.

### GST pulldown assays

Purified GST or GST-RIG-I-N proteins expressed in *E. coli* (BL21 cells) were conjugated to glutathione beads (GE Biosciences) at 4 °C for 2 . These beads were washed 5 times with lysis buffer and incubated with purified USP15 C250 (expressed in *E. coli* BL21 cells) at 4 °C for 6 h. After washing 5 times with lysis buffer, the beads were boiled in sample loading buffer and subjected to immunoblotting with indicated antibodies.

## Additional Information

**How to cite this article**: Zhang, H. *et al.* Ubiquitin-specific Protease 15 Negatively Regulates Virus-induced Type I Interferon Signaling via Catalytically-dependent and -independent Mechanisms. *Sci. Rep.* **5**, 11220; doi: 10.1038/srep11220 (2015).

## Supplementary Material

Supplementary Information
