# Supplementary material for: Ubiquitin-specific Protease 15 Negatively Regulates Virus-induced Type I Interferon Signaling via Catalytically-dependent and -independent Mechanisms
Source: Sci Rep. 2015 Jun 10;5:11220. doi: 10.1038/srep11220 (PMC4650652; doi:10.1038/srep11220)
Supplement: Supplementary Information [file srep11220-s1.pdf]

Ubiquitin-specific Protease 15 Negatively Regulates Virus-induced Type I Interferon Signaling via Catalytically-dependent and -independent Mechanisms

Huan Zhang, Dang Wang, Huijuan Zhong, Rui Luo, Min Shang, Dezhi Liu, Huanchun Chen, Liurong Fang*, Shaobo Xiao*

Supplementary Materials

Supplementary Figure S1. USP15 is involved in the regulation of the type I IFN signaling pathway in HEK293T cells.

Supplementary Figure S2. USP15 is involved in the regulation of the type I IFN signaling pathway in A549 cells.

Supplementary Figure S3. USP15 inhibits the RIG-I-N-induced activation of the IFN-β (a), ISRE (b), NF-κB (c) and IRF3 (d) promoters.

Supplementary Figure S4. Mutation of the catalytic residues does not completely abolish USP15 IFN antagonism.

Supplementary Figure S5. Comparison of IFN-regulation ability between USP15-HA and USP15-Myc.

Supplementary Table 1. Primers for effective genes of innate immunity used in real-time RT-PCR.

Supplementary Figure S1

a

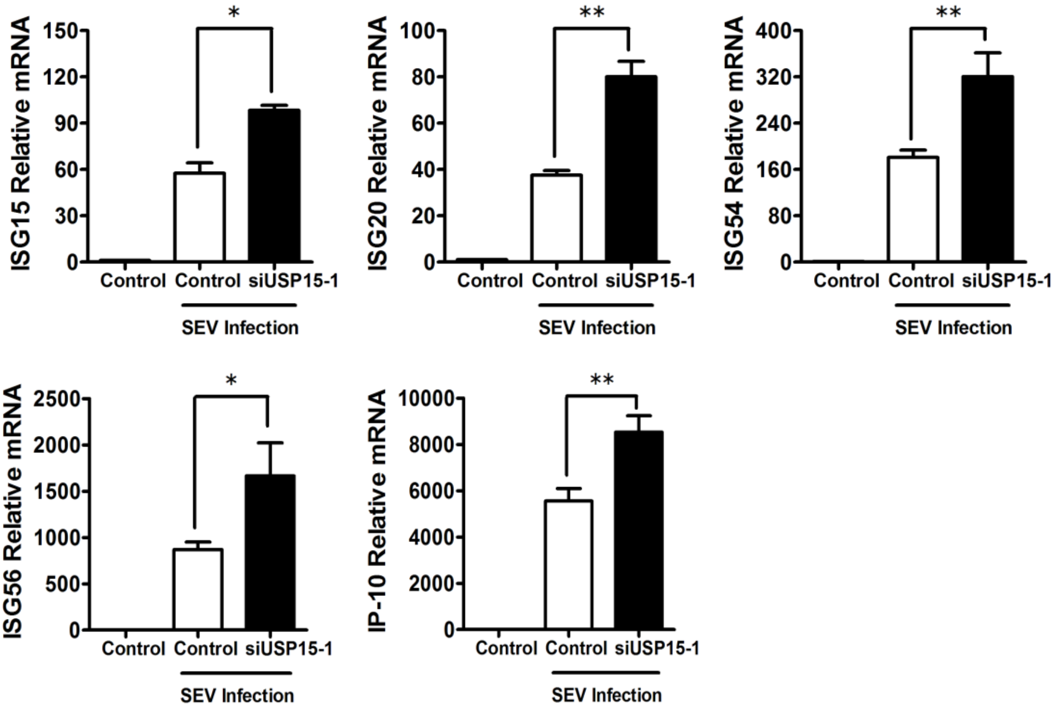

b

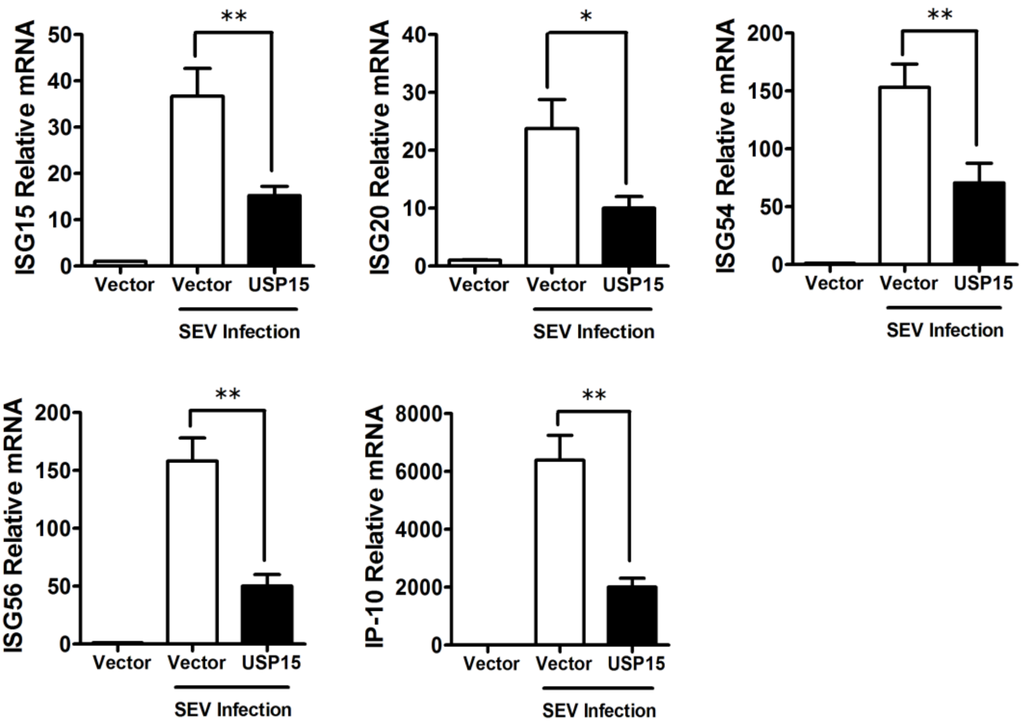

Figure S1. USP15 is involved in the regulation of the type I IFN signaling pathway in HEK293T cells. (a) Knockdown of USP15 upregulated the SEV-induced expression of ISGs in HEK293T cells. HEK293T cells were transfected with USP15-specific siRNAs or control siRNAs, 24 h later, the cells were infected with SEV or mock infected for 16 h. Total RNA was extracted and the expression of *ISG15* (57.6 vs 98.3, p=0.013), *ISG20* (37.5 vs 80.0, p=1.39E-03), *ISG54* (181.0 vs 320.7, p=1.05E-05), *ISG56* (870.2 vs 1665.6, p=0.019), and *IP-10* (5551.6 vs 8531.7, p=3.69E-03) was evaluated with SYBR Green real-time RT–PCR. Data are means ± SD from three independent experiments. ** indicates $p \leq 0.01$; * indicates $p \leq 0.05$. (b) USP15 inhibited the SEV-induced expression of ISGs. HEK293T cells were transfected with plasmid encoding USP15 (1 μg) or an equivalent amount of empty vector for 24 h, then the experiment was performed as in (a). *ISG15* (36.7 vs 15.2, p=6.77E-03), *ISG20* (23.8 vs 10.0, p=0.011), *ISG54* (153.1 vs 70.7, p=7.73E-04), *ISG56* (158.1vs 50.0, p=1.07E-04), and *IP-10* (6389.2 vs 2000.5, p=1.13E-03). Data are means ± SD from three independent experiments. ** indicates $p \leq 0.01$; * indicates $p \leq 0.05$.

Supplementary Figure S2

a

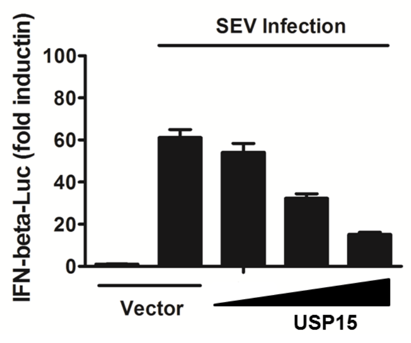

b

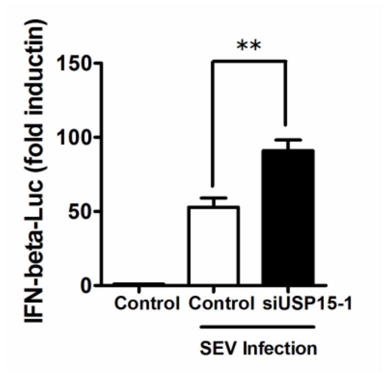

c

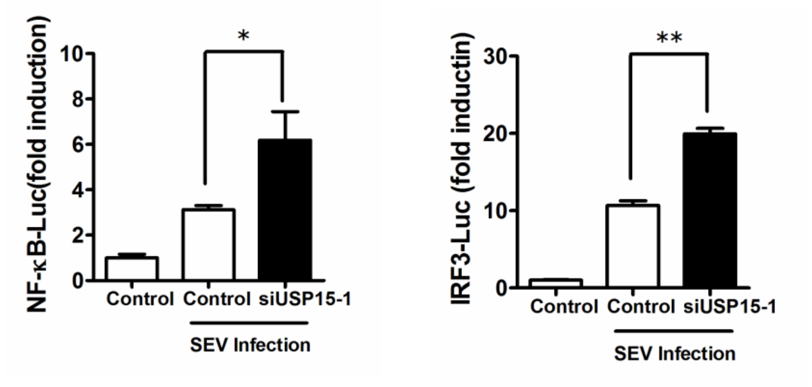

d

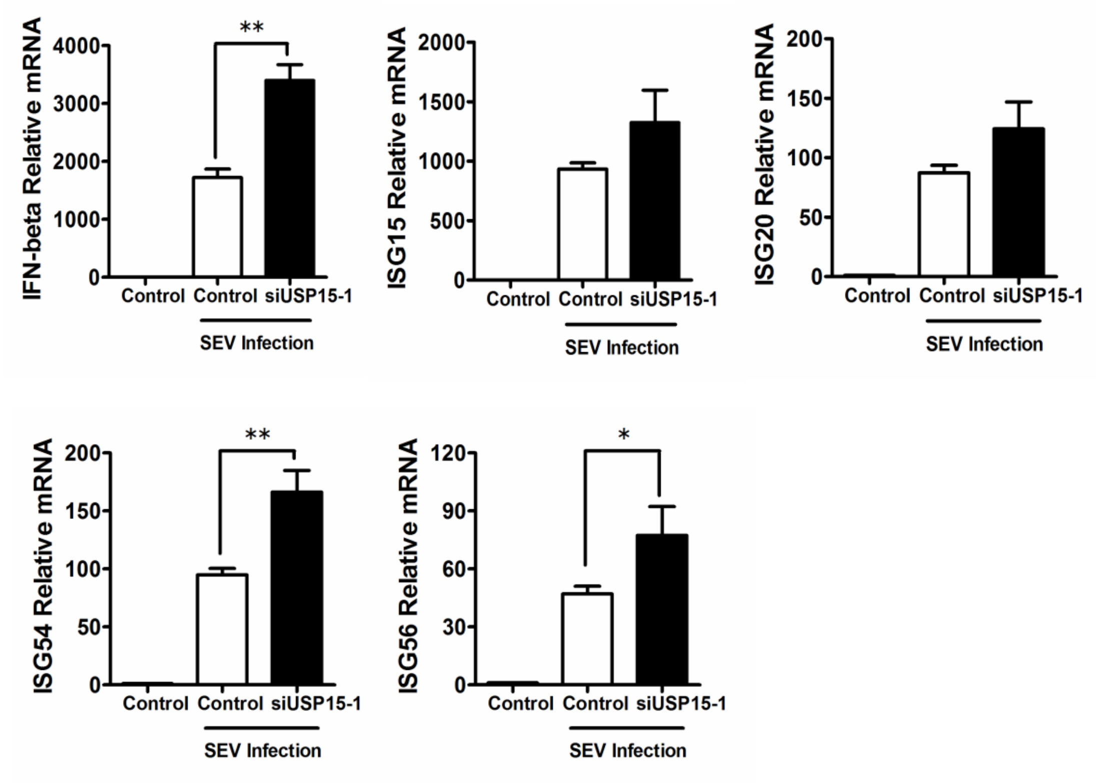

Figure S2. **USP15 is involved in the regulation of the type I IFN signaling pathway in A549 cells.** (a) USP15 inhibited the SEV-induced activation of the IFN-β promoter. A549 cells were transfected with IFN-β–Luc reporter plasmid (0.1 μg) and pRL-TK plasmid (0.02 μg), together with increasing amounts USP15 and then stimulated with SEV for 16 h before luciferase assay was performed. Data are means ± SD from three independent experiments. (b-c) A549 cells were transfected with different reporter plasmids and USP15-specific siRNAs and stimulated with SEV. The activity of IFN-β (52.9 vs 91.0, p=2.24E-03), NF-κB (3.1 vs 6.2, p=0.014) and IRF3 (10.7 vs 18.0, p=4.27E-06) was measured 16 h later. (d) Knockdown of USP15 upregulated the SEV-induced expression of *IFNB1* and ISGs in A549 cells. A549 cells were transfected with USP15-specific siRNAs or control siRNAs, 24 h later, the cells were infected with SEV or mock infected for 16 h. Total RNA was extracted and the expression of *IFNB1* (1722.6 vs 3399.7, p=6.99E-04), *ISG15* (934.1 vs 1324.8, p=0.071), *ISG20* (87.3 vs 124.2, p=0.053), *ISG54* (94.9 vs 166.3, p=3.17E-03) and *ISG56* (47.0 vs 77.4, p=0.027) was evaluated with SYBR Green real-time RT–PCR. Data are means ± SD from three independent experiments. ** indicates $p \leq 0.01$； * indicates $p \leq 0.05$.

Supplementary Figure S3

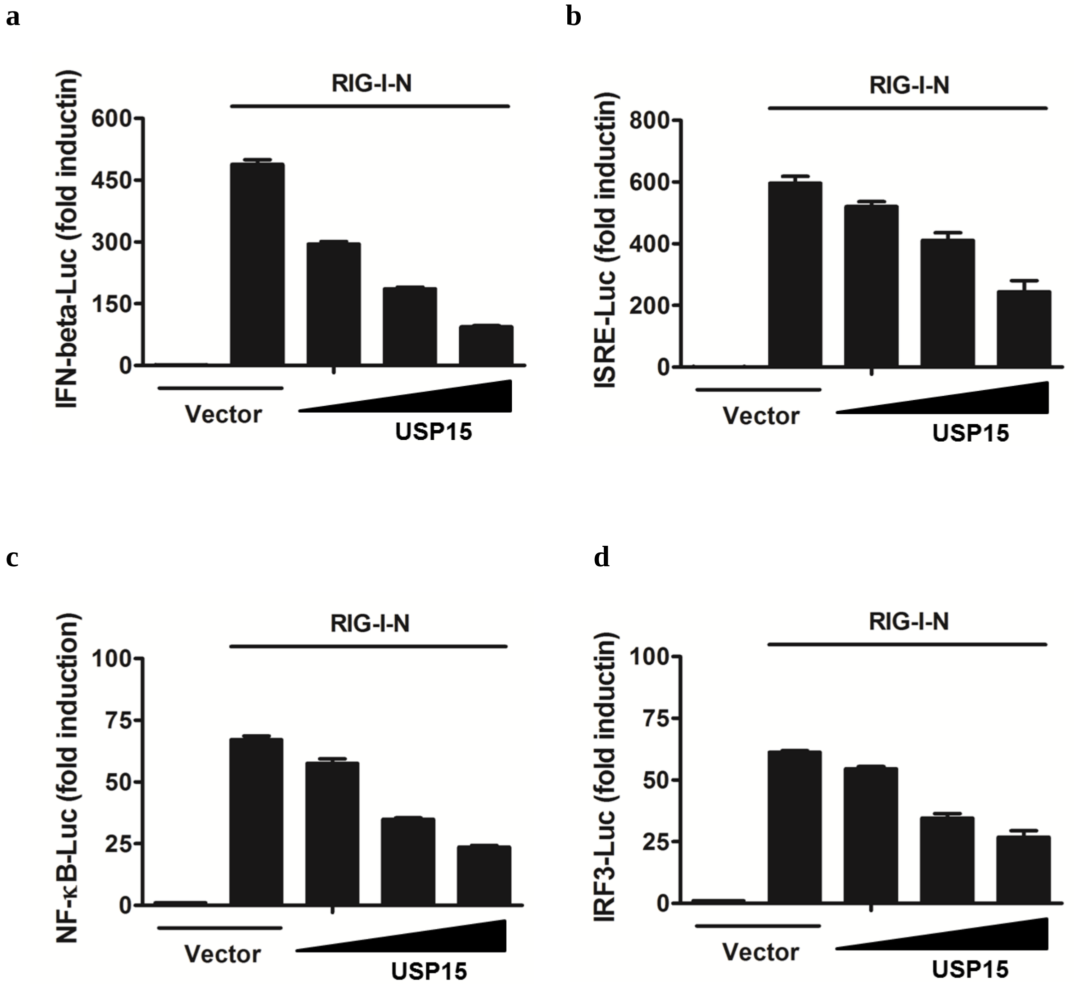

Figure S3. USP15 inhibits the RIG-I-N-induced activation of the IFN-β (a), ISRE (b), NF-κB (c) and IRF3 (d) promoters. HEK293T cells were transfected with indicated reporter plasmid (0.1 μg) and the pRL-TK plasmid (0.02 μg), together with increasing quantities (0, 0.15, 0.3, or 0.6 μg) of plasmid encoding USP15 and 0.4 μg of plasmid encoding RIG-I-N. Luciferase assays were performed 30 h after transfection. Data are means ± SD from three independent experiments.

Supplementary Figure S4

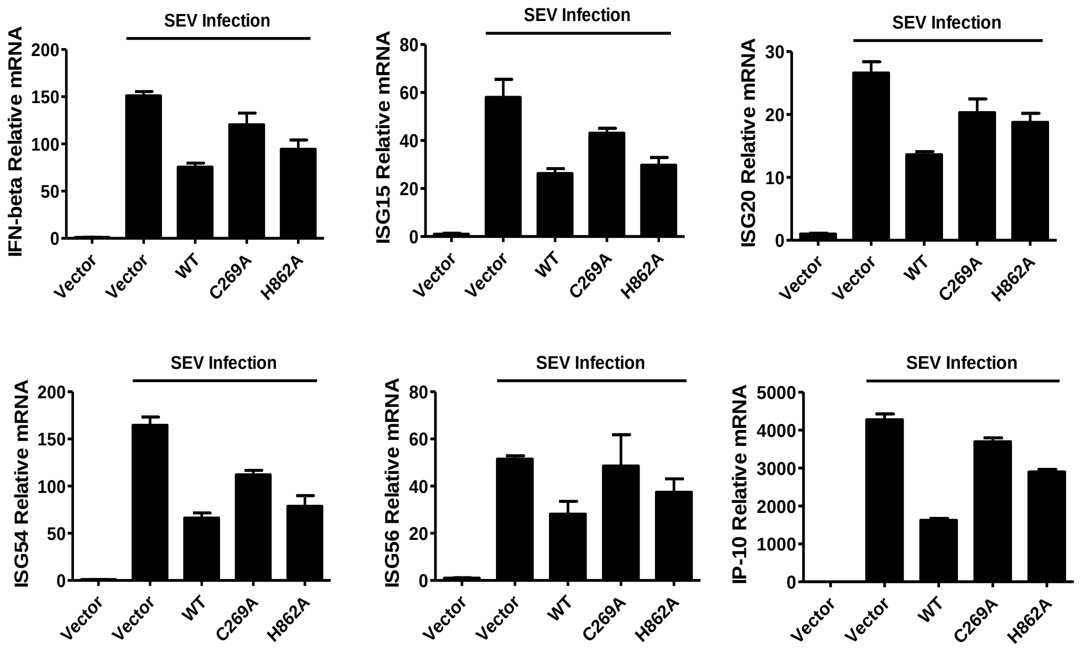

Figure S4. Mutation of the catalytic residues does not completely abolish USP15 IFN antagonism. HEK293T cells were transfected with plasmid encoding USP15, USP15 C269A, USP15 H862A (1 μg) or an equivalent amount of empty vector, 24 h later, the cells were infected with SEV or mock infected for 16 h. Total RNA was extracted and the expression of *IFNB1, ISG15, ISG20, ISG54, ISG56,* and *IP-10* was evaluated with SYBR Green real-time RT–PCR. Data are means ± SD from three independent experiments.

Supplementary Figure S5

a

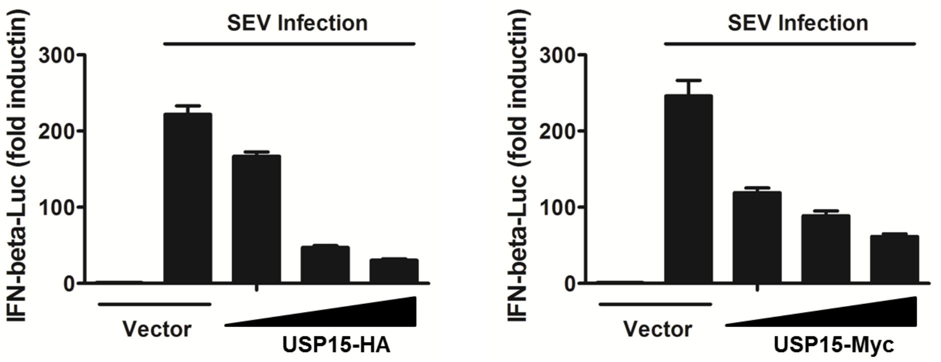

b

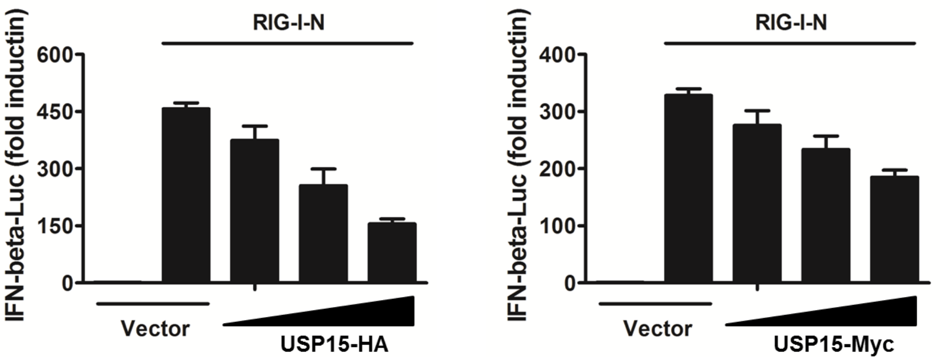

c

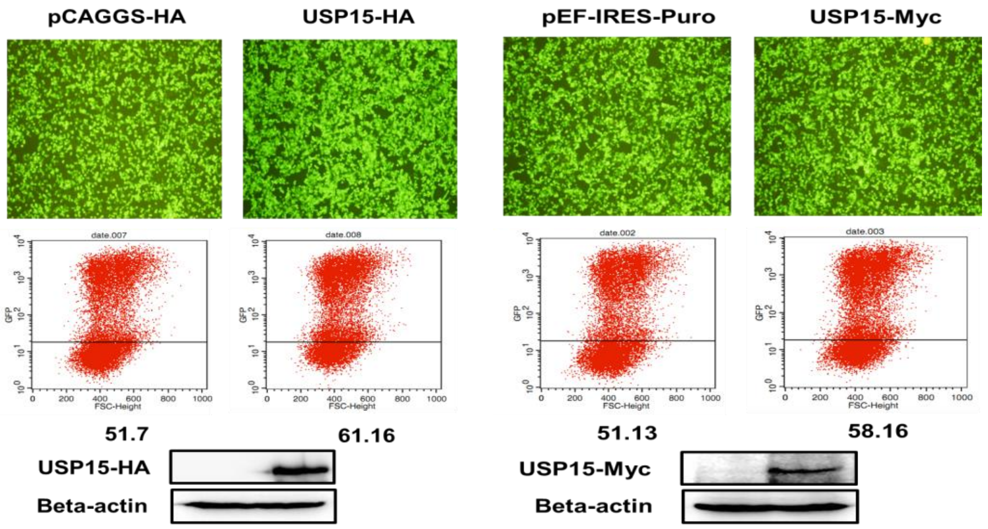

d

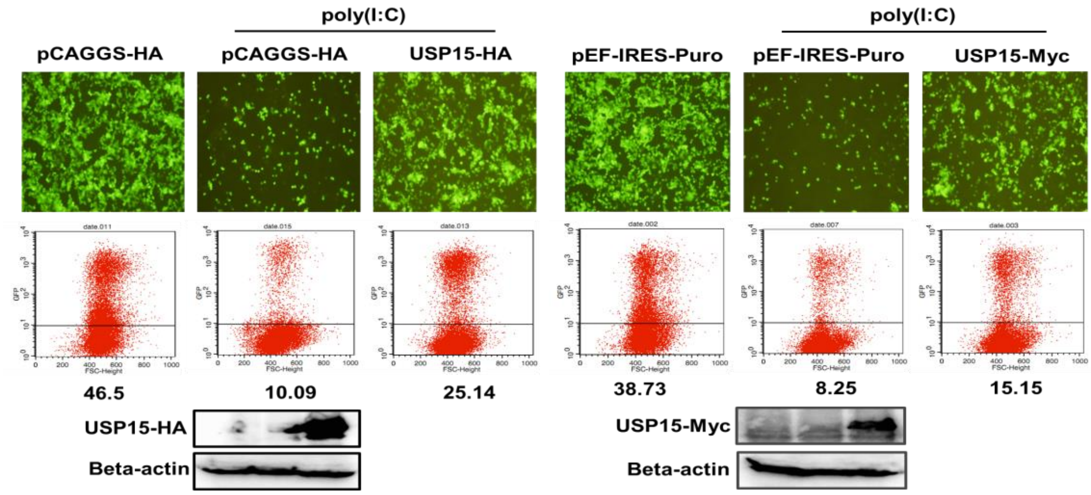

e

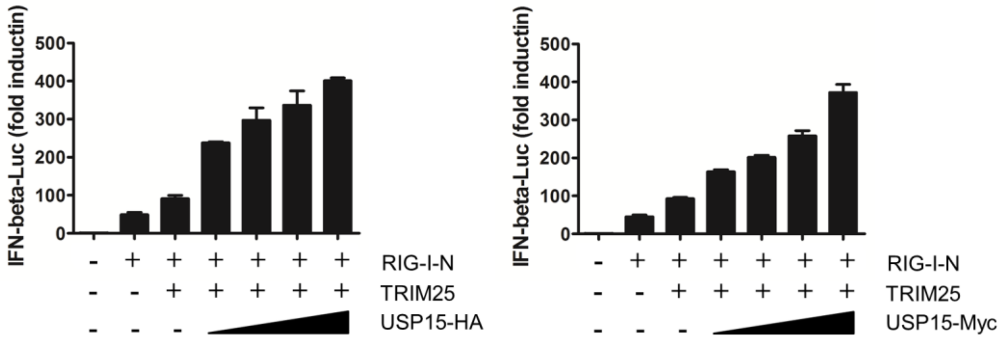

Figure S5. Comparison of IFN-regulation ability between USP15-HA and USP15-Myc. (a) USP15-HA and USP15-Myc inhibited the SEV-induced activation of the IFN-β promoter. HEK293T cells grown in 24-well plates were transfected with the IFN-β–Luc reporter plasmid (0.1 μg) and pRL-TK plasmid (0.02 μg) together with increasing quantities (0, 0.1, 0.3, or 0.9 μg) of plasmid encoding USP15-HA/USP15-Myc. At 24 h after transfection, the cells were further infected with SEV or mock infected for 16 h before luciferase assays were performed. Data are means ± SD from three independent experiments. (b) USP15-HA and USP15-Myc inhibited the RIG-I-N-induced activation of the IFN-β promoter. HEK293T cells were transfected with indicated reporter plasmid (0.1 μg) and the pRL-TK plasmid (0.02 μg),

together with increasing quantities (0, 0.15, 0.3, or 0.6 μg) of plasmid encoding USP15-HA/USP15-Myc and 0.4 μg of plasmid encoding RIG-I-N. Luciferase assays were performed 30 h after transfection. Data are means ± SD from three independent experiments. (c) HEK293T cells were transfected for 48 h with 4 μg USP15-HA/USP15-Myc plasmid, and then infected with VSV-GFP (MOI, 0.01). Fluorescence microscope was conducted 27 h post-infection and the rate of fluorescence was determined by flow cytometer. Western blotting analysis was performed to detect the USP15-HA/USP15-Myc proteins. (d) HEK293T cells were transfected for 24 h with USP15-HA/USP15-Myc and then further transfected with poly (I:C) (0.1μg) or left untreated for another 24h. HEK293T cells were incubated with the transfection supernatant for 24 h and infected with VSV-GFP (MOI, 0.01). Fluorescence microscope was conducted 27 h post-infection and the rate of fluorescence was determined by flow cytometer. Western blotting analysis was performed to detect the USP15-HA/USP15-Myc proteins. (e) USP15-HA and USP15-Myc enhanced TRIM25 and RIG-I-N induced IFN-β promoter. HEK293T cells were transfected with the IFN-β–Luc reporter plasmid (0.1 μg) and pRL-TK plasmid (0.02 μg) together with RIG-I-N and TRIM25 and increasing quantities of plasmid encoding USP15-HA/USP15-Myc. 48 h later, the luciferase assays were performed. Data are means ± SD from three independent experiments.

Supplementary Table 1. Primers for effective genes of innate immunity used in real-time RT-PCR.

RefSeq	symbol	Forward primer	Reverse primer
NM_002176	IFN-β	tctttccatgagctacaacttgct	gcagtattcaagcctcccattc
NM_005101	ISG15	gggacctgacggtgaagatg	cgccgatcttctgggtgat
NM_00201	ISG20	ccgtggccaggctagagat	ccgctcatgtcctctttcagt
NM_001547	ISG54	cacctctggactggcaatagc	gtcaggattcagccgaatgg
NM_001270927	ISG56	gctttcaaatcccttccgctat	gccttggcccgttcataat
NM_001565	IP-10	gtccacgtgttgagatcattgc	cctttccttgctaactgctttca
NM_002046	GAPDH	tcatgaccacagtccatgcc	ggatgaccttgcccacagcc
